# Attenuation of atherogenic apo B-48-dependent hyperlipidemia and high density lipoprotein remodeling induced by vitamin C and E combination and their beneficial effect on lethal ischemic heart disease in mice

**DOI:** 10.1186/s40659-018-0183-6

**Published:** 2018-09-15

**Authors:** S. Contreras-Duarte, P. Chen, M. Andía, S. Uribe, P. Irarrázaval, S. Kopp, S. Kern, G. Marsche, D. Busso, C. Wadsack, A. Rigotti

**Affiliations:** 1 Department of Nutrition, Diabetes and Metabolism, School of Medicine, Pontificia Universidad Católica de Chile, Diagonal Paraguay #362 - 4º, Piso, 8330024 Santiago, Chile; 2 Department of Radiology, School of Medicine, Pontificia Universidad Católica de Chile, Santiago, Chile; 3 Center of Molecular Nutrition and Chronic Diseases, School of Medicine, Pontificia Universidad Católica de Chile, Santiago, Chile; 4 Biomedical Imaging Center, School of Engineering, Pontificia Universidad Católica de Chile, Santiago, Chile; 5 Department of Electrical Engineering, School of Engineering, Pontificia Universidad Católica de Chile, Santiago, Chile; 6 Department of Obstetrics and Gynecology, Medical University of Graz, Graz, Austria; 7 Institute of Experimental and Clinical Pharmacology, Medical University of Graz, Graz, Austria

**Keywords:** HDL, Vitamin C and E, Serum lipids, Atherosclerosis

## Abstract

**Background and aims:**

Atherosclerotic cardiovascular disease is highly prevalent and its underlying pathogenesis involves dyslipidemia including pro-atherogenic high density lipoprotein (HDL) remodeling. Vitamins C and E have been proposed as atheroprotective agents for cardiovascular disease management. However, their effects and benefits on high density lipoprotein function and remodeling are unknown. In this study, we evaluated the role of vitamin C and E on non HDL lipoproteins as well as HDL function and remodeling, along with their effects on inflammation/oxidation biomarkers and atherosclerosis in atherogenic diet-fed SR-B1 KO/ApoER61^h/h^ mice.

**Methods and results:**

Mice were pre-treated for 5 weeks before and during atherogenic diet feeding with vitamin C and E added to water and diet, respectively. Compared to a control group, combined vitamin C and E administration reduced serum total cholesterol and triglyceride levels by decreasing apo B-48-containing lipoproteins, remodeled HDL particles by reducing phospholipid as well as increasing PON1 and apo D content, and diminished PLTP activity and levels. Vitamin supplementation improved HDL antioxidant function and lowered serum TNF-α levels. Vitamin C and E combination attenuated atherogenesis and increased lifespan in atherogenic diet-fed SR-B1 KO/ApoER61^h/h^ mice.

**Conclusions:**

Vitamin C and E administration showed significant lipid metabolism regulating effects, including HDL remodeling and decreased levels of apoB-containing lipoproteins, in mice. In addition, this vitamin supplementation generated a cardioprotective effect in a murine model of severe and lethal atherosclerotic ischemic heart disease.

## Background

Ischemic cardiovascular disease (CVD) is a leading cause of death worldwide. This disease is mainly due to atherosclerosis, and therefore is strongly associated to dyslipidemia, oxidative stress and inflammation [1]. Atherogenic dyslipidemia is mainly characterized by an increase in LDL and/or a decrease in HDL levels [2]. HDL particles are recognized as a protective factor against cardiovascular disease since they promote several atheroprotective functions [3] and their increment by 1 mg/dL is linked to an average decrease of 3% in the risk for development of CVD [4, 5]. During inflammation in acute phase or in chronic situations (e.g., different dysmetabolic diseases), HDLs undergo significant remodeling in their lipid as well as in their proteome content [6–10], resulting in impaired functions that lead to CVD [11]. Proatherogenic HDL dysfunctionality may include reduced antioxidation function [12–14]‚ impaired cholesterol efflux [7, 15], and reduced nitric oxide production [16].

Vitamins C and E have been described to protect LDL particles against oxidation [17] and their increased plasma concentrations are associated with reduced risk of stroke or coronary artery disease in humans [18, 19], likely through the ability of vitamin E to neutralize free radicals [20] and the capacity of vitamin C to scavenge reactive oxygen species and superoxide anion [21]. Furthermore, synergistic interaction between vitamins C and E may be critical at early stages of atherosclerotic lesion formation [22]. Indeed, previous work indicates that vitamin C and E co-administration attenuates atherosclerosis in double apo E/gulonolactone oxidase knockout mice [23]. Nevertheless, it has not been established if this vitamin combination may remodel HDL composition preventing or slowing down atherosclerosis progression when is administered before development of severe atherosclerosis that leads to ischemic complications and cardiac death.

Thus, the aim of this study was to evaluate the effect of preventive vitamin C and E supplementation on HDL composition and functionality, lipid metabolism, oxidation and inflammation biomarkers, atherosclerosis, and lifespan in atherogenic diet-fed SR-B1 KO/ApoER61^h/h^ mice.

## Materials and methods

### Mice

SR-B1^+/−^/ApoER61^h/h^ mice on mixed C57BL6J × 129Sv genetic background [24] were kindly provided by Dr. Monty Krieger. Homozygous SR-B1 KO/ApoER61^h/h^ mice were obtained through SR-B1^+/−^/ApoER61^h/h^ intercrosses followed by offspring genotyping.

### Animal procedures

Male and female SR-B1 KO/ApoER61^h/h^ littermates had free access to normal chow diet [Prolab RMH 3000 (22% protein, 5% fat and 0.02% cholesterol) obtained from PMI Feeds, Inc., Brentwood, CA, USA] and water until use for experiments.

During vitamin pre-treatment for 5 weeks before atherogenic diet feeding, SR-B1 KO/ApoER61^h/h^ mice received water supplemented with 1.7 mg vitamin C/50 mL as well as chow diet with 2000 IU vitamin E/kg. These vitamin doses were chosen based on previous reports that indicated significant effects on oxidative stress, various biomarkers, and atherogenesis in other murine models of atherosclerosis [23, 25, 26]. We used a vitamin C and E combination approach to preserve an active antioxidant regenerating system in our mouse model [27, 28].

At the end of this pre-atherogenesis phase, chow diet was replaced by atherogenic diet [Test Diet 57BB (20.6% protein, 15% fat, 1.25% cholesterol and 0.5% sodium cholate) obtained from Lab Diet, St. Louis, MO, USA] maintaining vitamin addition in water and diet. A control group was fed with atherogenic diet without vitamin supplementation. Some control and vitamin C and E-treated mice were euthanized after 10 days of atherogenic diet feeding to collect serum and tissue samples, which were stored at − 80 °C until analyses. Hearts were frozen in cryopreserving liquid and stored at − 80 °C for histological studies. Additional control and vitamin C and E-treated mice were kept with atherogenic diet feeding until death.

All procedures were evaluated and approved by the Animal Care and Wellbeing Committee at the School of Medicine, Pontificia Universidad Católica de Chile.

### Lipoprotein fractionation and high density lipoprotein isolation

Serum was fractionated by fast-protein liquid chromatography (FPLC) and apolipoprotein A-I-containing fractions were pooled and concentrated using Amicon Ultra-4 centrifugal 10 K filters (Millipore, Merck, Darmstadt, HD, Germany). Pooled fractions were subjected to dynabead-based immunoprecipitation with an anti-apolipoprotein B (apo B) antibody to remove apo B-containing lipoproteins. Apo B lipoprotein-depleted supernatants were subjected to discontinuous density gradient ultracentrifugation to isolate high density lipoproteins (1.063 < d < 1.21 g/mL). After centrifugation, HDL fractions were collected, desalted via PD10 columns (GE Healthcare, Vienna, WI, Austria), and immediately used for experiments.

### Total serum and HDL lipid and protein levels

Total serum and HDL lipids were measured using enzymatic kits from DiaSys Diagnostic Systems GmbH (Holzheim, BY, Germany). Total unesterified cholesterol in serum samples was determined by an enzymatic kit from Sigma Aldrich (St. Louis, MO, USA). Total HDL protein content was measured using BCA kit from Thermo Fischer Scientific Inc. (Waltham, MA, USA).

### Total serum apolipoprotein B-100 levels

Serum apolipoprotein B-100 (apo B-100) levels were measured using Mouse Apolipoprotein B (APOB) ELISA (Cusabio Biotech Co. Ltd., Wuhan, China) following commercial recommendations.

### Serum and HDL protein immunoblotting

1 µL of serum or 15 µg of total HDL protein were subjected to SDS-PAGE using 4–20% acrylamide gradient gels (Thermo Scientific, Rockford, IL, USA), electrotransferred and immunoblotted with monoclonal anti-paraoxonase 1 (PON1) (1:2000 dilution), polyclonal anti-apolipoprotein A-I (apo A-I) (1:2000 dilution), and anti-apolipoprotein B-48 (apo B-48) (1:2000 dilution) antibodies from Abcam (Cambridge, UK) as well as polyclonal anti-apolipoprotein D (apoD) (1:200 dilution) and anti-phospholipid transfer protein (PLTP) (1:500 dilution) antibodies from Novus Biological Co. (Littleton, CO, USA). Protein/antibody complexes were detected with HRP-conjugated secondary antibodies. HRP activity was detected using Pierce ECL Western Blotting substrates (Thermo Scientific, Rockford, IL, USA). Images were analyzed with Image J software.

### Phospholipid transfer protein activity

Lipid transfer assay for phospholipid transfer protein (PLTP) was performed in total serum according to manufacturer’s protocols for a PLTP kit (Biovision Inc., Milpitas, CA, USA).

### Serum cytokine levels

Serum TNF-α, IL-1β, IL-6 and IL-10 measurements were performed using mouse TNF-alpha Quantikine ELISA, IL-1 beta Quantikine ELISA, IL-6 Quantikine ELISA, and IL-10 DuoSet ELISA kits (R&D Systems, Minneapolis, MN, USA) according to commercial recommendations.

### HDL anti-oxidant capacity

Anti-oxidant function of HDL was determined as reported elsewhere [29, 30] by measuring inhibition of dihydrorhodamine (DHR) oxidation in vitro. 10 μg of HDL protein and 15 μL of DHR solution were added and final volume was completed with HEPES buffer to 100 μL. Increase in fluorescence due to the oxidation of DHR was measured every 2 min for 1 h at 485 excitation/538 nm emission. HDL anti-oxidant activity was calculated from kinetic slopes of DHR oxidation and expressed as percentage of slope values obtained from assays without addition of HDL samples.

### Histological analysis

Serial 10 μm cryosections from aortic roots were stained for neutral lipids with Oil Red O. Images were obtained using a Nikon eclipse E200 microscope with a 4X objective and NIS-*Element* F3.2 Viewer program. Atheromatous plaque areas were quantified at aortic roots, in which the three valve leaflets were present. Results were expressed as cross sectional lesion area/total aortic area ratios.

### Survival studies

Atherogenic diet-fed mice with and without vitamin C and E treatment were evaluated daily and monitored until death. Survival data were tabulated and expressed by Kaplan–Meier curves as function of time after initiation of the atherogenic diet feeding.

### Statistical analysis

Data were analyzed using unpaired Student’s t-test or Mann Whitney test according to results obtained in normality tests and log-rank analysis was performed for survival experiments (GraphPad Prism, La Jolla, CA, USA). Differences observed between control and treated mice were considered statistically significant when *P *< 0.05.

## Results

**Fig. 1:**
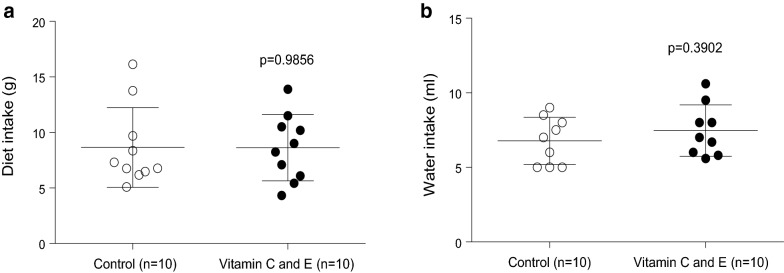
Daily diet (**a**) and water (**b**) intake in SR-B1 KO/ApoER61^h/h^ mice fed with atherogenic diet. Data were obtained from two independent animal sets

In order to ensure that the effects of the vitamin combination (see below) were not due to changes in intake of the atherogenic diet, food and water intake were measured in control and vitamin C and E supplemented mice. No significant changes in food intake or water drinking were observed between both groups (Fig. 1a, b).

To evaluate the effect of vitamin C and E supplementation on serum lipids of atherogenic diet-fed SR-B1 KO/ApoER61^h/h^ mice, total and unesterified cholesterol, triglycerides and phospholipid levels were measured. All serum lipids were significantly decreased in vitamin C and E-treated mice compared to the control group (Table 1).

**Table 1:** Effect of vitamin C and E on serum lipids in SR-B1 KO/ApoER61^h/h^ mice fed with atherogenic diet

Serum lipids	Atherogenic diet-fed SR-BI KO/ApoER61^h/h^ mice		
	Control (n = 26)	Vitamin C + E (n = 24)	p value
Total cholesterol (mg/dL)	1080 ± 292	619 ± 168	< 0.0001
Unesterified cholesterol (UC) (mg/dL)	708 ± 294	354 ± 105	< 0.0001
UC/total cholesterol ratio	0.675 ± 0.30	0.574 ± 0.22	0.1944
Triglycerides (mg/dL)	55 ± 32	28 ± 9	0.0008
Phospholipids (mmol/L)	0.172 ± 0.08	0.097 ± 0.04	0.0003

Values are shown as mean ± standard deviation. Lipids were measured from serum samples obtained 10 days after the beginning of atherogenic diet consumption

**Fig. 2:**
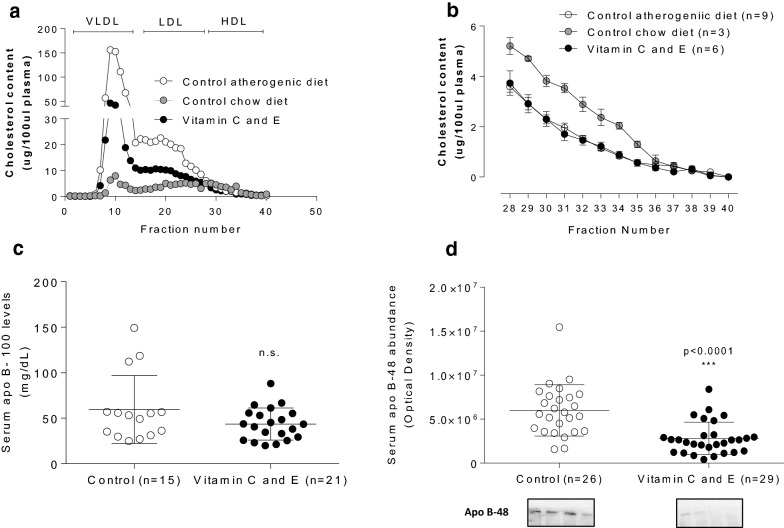
Effect of vitamin C and E on lipoprotein cholesterol profile and serum abundance of apolipoproteins in SR-B1 KO/ApoER61^h/h^ mice fed with atherogenic diet. **a** Overall lipoprotein cholesterol profile. **b** HDL FPLC fractions. Serum abundance of **a** apolipoprotein B-100 and **b** apolipoprotein B-48. Experimental data were obtained from two independent animal sets in A and four independent sets in C and D. Samples were obtained 10 days after the beginning of atherogenic diet consumption. n.s.: p not significant

To determine which lipoprotein changes explained the vitamin-induced hypocholesterolemic effect, serum fractionation was performed and lipoprotein cholesterol levels were measured in each fraction from control and vitamin C and E-treated mice after feeding atherogenic diet. Vitamin C and E combination reduced cholesterol transported mainly in large VLDL-sized lipoproteins as well as in IDL/LDL range particles in atherogenic diet-fed SR-B1 KO/ApoER61^h/h^ mice (Fig. 2a), without significant effect in cholesterol content of normal HDL-sized lipoproteins even it is lower than chow-fed SR-B1 KO/ApoER61^h/h^ mice (Fig. 2b). Since diminished triglycerides and decreased cholesterol content in large lipoproteins were found, serum abundance of apo B-100 and apo B-48 were measured. Whereas apo B-100 levels were not changed compared to control mice (Fig. 2c), vitamin C and E administration significantly decreased apo B-48 abundance (Fig. 2d).

**Fig. 3:**
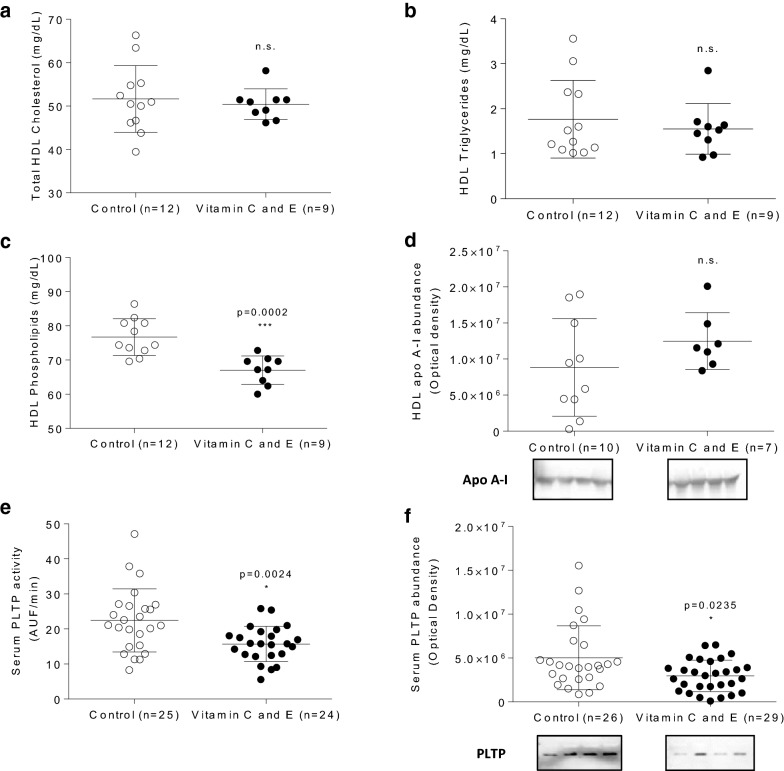
Effect of vitamin C and E combination on HDL lipids and apo A-I and serum phospholipid transfer protein in SR-B1 KO/ApoER61^h/h^ mice fed with atherogenic diet. **a** HDL cholesterol. **b** HDL triglycerides. **c** HDL phospholipids. **d** Apolipoprotein A-I abundance. **e** Phospholipid transfer protein (PLTP) activity. **f** PLTP abundance. Experimental data were collected from four independent animal sets. Samples were obtained 10 days after the beginning of atherogenic diet consumption. n.s.: p not significant

To evaluate the effect of vitamin C and E on HDL lipids, these lipoproteins were isolated and their lipids were measured. Isolated HDL from vitamin-supplemented animals did not exhibit changes in total cholesterol and triglyceride content or in apo A-I, the main HDL apolipoprotein (Fig. 3a, b, d). However, vitamin C and E use in this murine model led to a significant decrease in HDL phospholipid levels expressed as absolute levels (Fig. 3c) or adjusted by total HDL lipid content (56.3 ± 2.6 versus 59.1 ± 3.9 mg/100 mg total HDL lipids for vitamin C and E- and control-treated mice, respectively). This lower HDL phospholipid content was most likely due the diminished abundance and activity of phospholipid transfer protein (PLTP) observed in vitamin C and E-treated animals compared to controls (Fig. 3e, f).

**Fig. 4:**
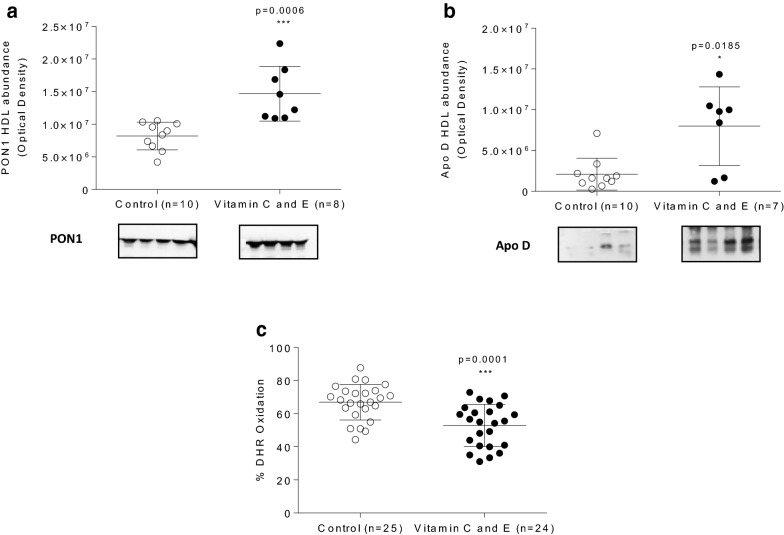
Effect of vitamin C and E combination on HDL paraoxonase-1 and apolipoprotein D levels and HDL antioxidant capacity in SR-B1 KO/ApoER61^h/h^ mice fed with atherogenic diet. **a** HDL paraoxonase-1 abundance. **b** HDL apolipoprotein D abundance. **c** Antioxidant HDL capacity. Experimental data were collected from four independent animal sets. Samples were obtained 10 days after the beginning of atherogenic diet consumption

Since HDL display antioxidant functions, the content of PON1 and apo D, two critical HDL proteins with known antioxidant functions, was further evaluated. The abundance of HDL PON1 and apo D were significantly increased by vitamin use in this murine model (Fig. 4a, b), which is consistent with the increased HDL antioxidant capacity found in vitamin C and E-treated mice (Fig. 4c).

**Fig. 5:**
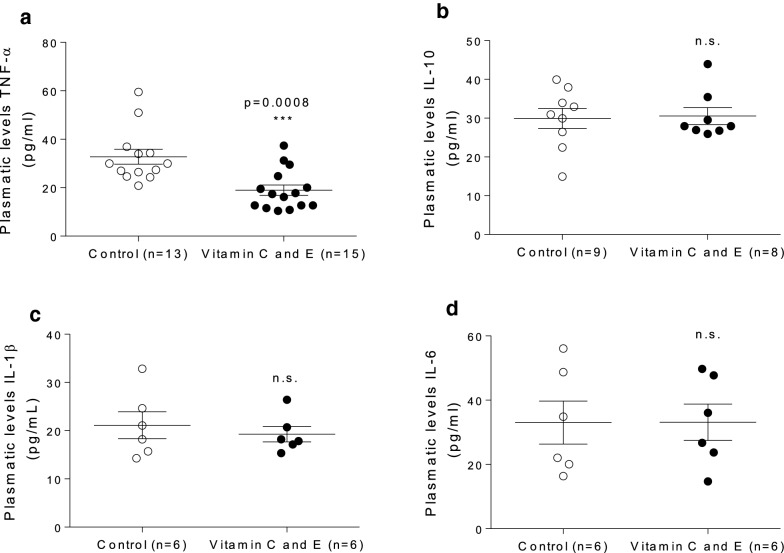
Effect of vitamin C and E combination on plasma levels of pro- and anti-inflammatory cytokines in SR-B1 KO/ApoER61^h/h^ mice fed with atherogenic diet. **a** TNF-α. **b** IL-10. **c** IL-1β. **d** IL-6. Experimental data were collected from four independent animal sets. Samples were obtained 10 days after the beginning of atherogenic diet consumption. n.s.: p not significant

In addition, a significant decrease in levels of TNF-α, a pro-inflammatory cytokine, in vitamin-supplemented mice was detected relative to the control group (Fig. 5a), without significant changes in IL-1β, IL-6, or IL-10 (Fig. 5 b–d).

**Fig. 6:**
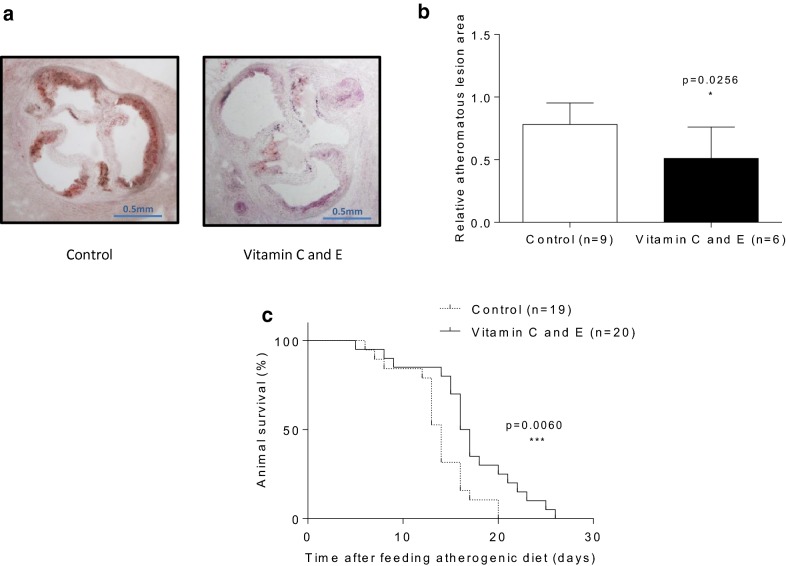
Effect of vitamin C and E combination on aortic root atherosclerosis and lifespan in SR-B1 KO/ApoER61^h/h^ mice fed with atherogenic diet. **a** Atheromatous neutral lipid staining. **b** Atheromatous lesion quantification. **c** Survival curve. Experimental data were collected from three independent animal sets. For A and B, samples were obtained 10 days after the beginning of atherogenic diet consumption

Finally, decreased atheromatous lesion size with reduced luminal stenosis at aortic root levels were found in atherogenic diet-fed SR-B1 KO/ApoER61^h/h^ mice supplemented with vitamin E and C (Fig. 6a, b). With regard to lifespan, median survival time of control mice (14 days) was increased to 16.5 days (18% survival time extension (p = 0.006)) by vitamin administration in atherogenic diet-fed SR-BI KO/ApoER61^h/h^ mice (Fig. 6c).

## Discussion

This study reports that preventive vitamin C and E supplementation leads to anti-atherogenic HDL remodeling and functioning along with decreased levels of apoB-containing lipoproteins and attenuated coronary disease in atherogenic diet-fed SR-BI KO/ApoER61^h/h^ mice.

Vitamins C and E remodeling effects included a reduction in HDL phospholipid content accompanied by decreased PLTP abundance and activity, which are most likely leading to HDL particles with improved atheroprotection. Besides lipid remodeling, these vitamins promoted additional HDL protein modifications, increasing components such as PON1 and apo D, which correlated with improved HDL antioxidant activity. Despite of a slight decrease in levels of HDL sized particles in the murine model of atherosclerosis used in this study (compared to mice with the same genetic background but fed with chow diet), the remaining presence of these HDL particles in atherogenic diet-fed mice are most likely acting as antiatherogenic agents, particularly after the induction of beneficial changes in HDL protein composition and function due to vitamin C and E treatment. These changes possibly indirectly decreased serum levels of the inflammatory biomarker TNF-α. Since vitamin C and E supplementation also reduced cholesterol levels transported in apo B-containing lipoproteins, attenuation of this proatherogenic dyslipidemia may have played a role in reducing atherogenesis and increasing lifespan in atherogenic diet-fed SR-B1 KO/ApoER61^h/h^ mice, a known murine model of severe dyslipidemia, progressive atherosclerosis, coronary heart disease and premature death. In this study, all biological samples were taken and measurements were performed during atherogenic diet feeding associated with vitamin supplementation. However, similar analyses at the end of pre-treatment with vitamins before induction of atherogenesis would have been very informative in order to better understand the effect of vitamin C and E combination alone.

There is no previous report of a selective reduction in HDL phospholipids levels as a consequence of vitamin C and/or E supplementation. Remarkably, vitamin C and E treatment also diminished PLTP abundance and activity. In this regard, it is well known that PLTP remodels and transforms HDL3 into HDL2 through phospholipid transfer from chylomicrons and VLDL, therefore regulating the HDL particle size [30]. Reduced PLTP due to vitamin supplementation may explain the decreased content of HDL phospholipids, generating smaller HDL particles with known atheroprotective function [31–33]. Additionally, it is well established that overexpression of PLTP increases atherosclerosis [34, 35] and decreased expression, depending on the pathophysiological context, may protect against atherosclerosis [36, 37] as found in this study.

Vitamin C and E are well-established antioxidant agents in vitro and in vivo [17, 20]. This activity was confirmed in our work by the improvement of the HDL antioxidant function most likely due to HDL lipid and protein remodeling. This is the first time that vitamin C and/or E have been linked to HDL antioxidant remodeling, particularly in apo D and PON1 content, compared to the control group. Remarkably, these modified HDL particles exhibited higher antioxidant capacity, suggesting an improved atheroprotective function. Previous work has shown that probucol, an antioxidant biomolecule, also exhibits an anti-atherogenic effect in double SR-B1/apoE KO mice [38], another model of lethal coronary heart disease very similar to atherogenic diet-fed SR-B1 KO/ApoER61^h/h^ mice used in this investigation. Probucol restored normal lipoprotein distribution, HDL cholesterol levels, and unesterified/total HDL cholesterol ratio in double KO mice [38], however no additional analyses were performed on HDL protein and function -including its antioxidant capacity- even though probucol is considered a vitamin E analog with antioxidative properties. Thus, our findings are novel regarding the impact of vitamin C and E combination on HDL particles beyond their cholesterol content in atherogenic diet-fed SR-B1 KO/ApoER61^h/h^ mice. However, vitamin E supplementation may have also led to a higher content of tocopherol in HDL particles, which *per se* may be contributing to the improved HDL antioxidant capacity found after vitamin C and E feeding in this atherosclerosis prone murine model. Indeed, previous work showed that vitamin E feeding at the same dose used in our study (2000 I.U. α-tocopherol/kg diet) in female SR-B1 +/− mice increased lipoprotein vitamin E levels [39]. Further analysis of vitamin E content in HDL particles should be performed in vitamin E supplemented atherogenic diet-fed SR-B1 KO/ApoER61h/h mice to address this issue.

The use of the vitamin C and E combination in this experimental model also determined an important effect on blood lipids, characterized by a significant overall decrease in serum levels of total (esterified and unesterified) cholesterol, triglycerides, and phospholipids. This effect does not seem explained by decreased atherogenic diet intake (Fig. 1a). Previous studies have revealed that vitamin E treatment can reduce plasma cholesterol levels in animal models of atherosclerosis [23]. The effect of vitamin C and E combination on apo B-48, but not apoB-100, levels suggests a specific impact on apo B-48-dependent lipoprotein production and/or catabolism, rather than an overall effect on apo B-containing lipoprotein metabolism. Indeed, vitamin C inhibits acyl-coA:cholesterol acyltransferase (ACAT), a key intracellular enzyme involved in intestinal chylomicron assembly as well as hepatic VLDL synthesis [40]. This vitamin also seems to reduce 3-hydroxy-3-methylglutaryl coenzyme A (HMG-CoA) reductase activity, which controls the rate-limiting step during cholesterol biosynthesis [40], leading to decrease in hepatic cholesterol synthesis, compensatory increase in LDL receptor levels, enhanced receptor-mediated lipoprotein catabolism, and reduction in circulating levels of apo B-containing lipoproteins [41]. On the other hand, in vitro studies have shown that vitamin C inhibits post-translational processing of SREBP-2 transcription factor, which reduces HMG-CoA reductase expression, therefore decreasing cholesterol synthesis and availability for lipoprotein assembly and increasing apo B-48 lipoprotein clearance [41].

In addition, we cannot rule out that changes observed in non-HDL lipoproteins due to vitamin C and E supplementation in atherogenic diet-fed SR-B1 KO/ApoER61^h/h^ mice also had a significant role in modulating HDL composition and function. Indeed, reduced levels of apo B-48 containing lipoproteins are very likely leading to decreased non HDL phospholipid availability, which associated with reduced PLTP activity and abundance, may have determined less transfer of phospholipids from chylomicrons and VLDL to HDL and, consequently, reduced HDL phospholipid content.

Reduced oxidative stress and increased antioxidant defense attenuate pro-inflammatory response. We found lower levels of TNF-α in animals treated with vitamin C and E. It is known that vitamin C suppresses TNF-α activation mediated by NF-kB in cardiomyocytes [42], which allow us to hypothesize that this vitamin may have also induced a primary effect on inflammation beyond improvement of anti- versus pro-oxidative balance.

Based on reduced apo B lipoprotein cholesterol levels, HDL phospholipid and protein remodeling, increased antioxidant HDL function, and reduced inflammation, we predicted reduced atherosclerosis after vitamin C and E administration in atherogenic diet-fed SR-B1 KO/ApoER61^h/h^ mice. Indeed, these vitamin-treated animals showed significant reduction in atheromatous load and stenosis in the aortic root. Previous studies in other vitamin C or E-treated murine models support our findings [42, 43]. Furthermore, double apo E/gulonolactone oxidase knockout mice treated with vitamin C improved atheromatous plaque morphology without changes in plaque sizing [42]. In addition, apo E-deficient mice exhibited reduced aortic atheromatous lesions [43]. Moreover, vitamin C and E-deficient apo E/gulonolactone oxidase knockout mice were used to evaluate the effect of vitamin supplementation on atherosclerosis. After vitamin C and E treatment during 8 weeks, lowered oxidative and inflammatory biomarkers and reduced atherosclerotic plaque size were observed in this mouse model [23].

Increased lifespan induced by vitamin C and E pre-treatment in atherogenic diet-fed SR-B1 KO/ApoER61^h/h^ mice may be explained by reduced levels of atherogenic lipoproteins and improved intermediate metabolic biomarkers linked to oxidative stress and inflammation together with atheroprotective HDL remodeling, which led to reduced atherosclerosis progression with delayed fatal ischemic complications due to coronary heart disease. Previous studies have shown that ezetimibe [44], intestinal bile salt absorption inhibitor [44], and pomegranate juice [45] can attenuate the spontaneous severe and fatal phenotype of double SR-B1/apoE KO mice. Our work is the very first report of reduced atherosclerosis and increased lifespan due to vitamin C and E administration in this severe murine model of CVD.

Whether these findings can be translated to humans remains to be established due to the severe and rapidly progressive atherosclerotic disease observed in these mice. However, atherogenic diet-fed SR-B1 KO/ ApoER61^h/h^ or double SR-B1/apoE KO mice represent a valuable tool considering that not many models of coronary heart disease and its ischemic complications, including heart failure and death, are available. Indeed, these mouse models of occlusive coronary atherosclerosis appear to develop features that are clinically relevant for human disease and are useful to evaluate natural and drug compounds that modulate cholesterol homeostasis and atherosclerosis as mentioned above, and thus seem to be pertinent for translational cardiovascular research from animals to humans.

## Conclusions

This study shows that preventive vitamin C and E supplementation leads to protective HDL remodeling, increased antioxidant HDL protein content and improved HDL antioxidant activity as well as attenuated atherogenic apo B-48-dependent hyperlipidemia and reduced inflammation in atherogenic diet-fed SR-B1 KO/ApoER61^h/h^ mice. Taken together, these findings may explain its beneficial impact against atherosclerosis, leading to reduced myocardial infarction and increased survival observed in this murine model of severe and lethal ischemic coronary heart disease.
